# Targeted Therapy of Ewing's Sarcoma

**DOI:** 10.1155/2011/686985

**Published:** 2010-10-31

**Authors:** Vivek Subbiah, Pete Anderson

**Affiliations:** Department of Pediatrics, The University of Texas, MD Anderson Cancer Center, 1515 Holcombe Boulevard, Houston, TX 77030, USA

## Abstract

Refractory and/or recurrent Ewing's sarcoma (EWS) remains a clinical challenge because the disease's resistance to therapy makes it difficult to achieve durable results with standard treatments that include chemotherapy, radiation, and surgery. Recently, insulin-like-growth-factor-1-receptor (IGF1R) antibodies have been shown to have a modest single-agent activity in EWS. Patient selection using biomarkers and understanding response and resistance mechanisms in relation to IGF1R and mammalian target of rapamycin pathways are areas of active research. Since EWS has a unique tumor-specific EWS-FLI1 t(11;22) translocation and oncogenic fusion protein, inhibition of EWS-FLI1 transcription, translation, and/or protein function may be key to eradicating EWS at the stem-cell level. Recently, a small molecule that blocks the protein-protein interaction of EWS-FLI1 with RNA helicase A has been shown in preclinical models to inhibit EWS growth. The successful application of this first-in-class protein-protein inhibitor in the clinic could become a model system for translocation-associated cancers such as EWS.

## 1. Introduction

**Figure 1:**
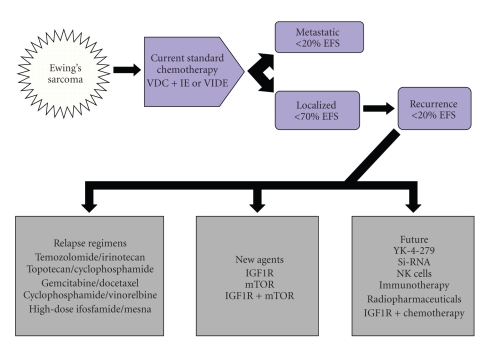
Current and future EWS treatment options. Current treatment of EWS typically employs VAC+IE or VIDE regimens. Local control includes surgery and/or radiation therapy. With this regimen, patients with only local disease have about 70% disease-free survival (EFS). However, patients with EWS who have metastatic disease or who have recurrence have <20% EFS. Second-line relapse regimens as shown below often provide temporary benefit. New agents against IGF1R and /or mTOR are currently available. Future options include innovative targeted therapies. V: vincristine; D: doxorubicin; C: cyclophosphamide; I: ifosphamide/mesna; E: etoposide; IGF1R insulin-like growth factor 1 receptor inhibitor; mTOR: mammalian target of rapamycin inhibitor.

**Figure 2:**
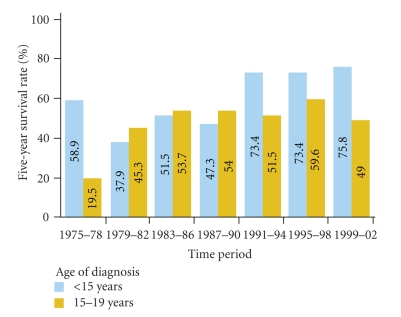
Five-year survival rates for Ewing's sarcoma (EWS), 1975–2006. The 5-year survival rates for EWS among children and adolescents is shown by age group and time period of diagnosis from 1975 through 2002, with follow-up of survival through 2006; data are from the Surveillance, Epidemiology, and End Results 9 (SEER 9) registries (Reprinted with permission from Smith et al. J Clinical Onc 2010; 28:2625-2634).

In patients with localized Ewing's sarcoma (EWS), the standard 5-drug cytotoxic chemotherapy regimen administered by the Children's Oncology Group (COG) results in a disease-free survival rate of 60%–70%. In EWS patients with metastatic or recurrent disease, however, the same 5-drug regimen results in a disease-free survival rate of less than 20%, which is not improved with chemotherapy intensification or stem cell transplantation [1, 2] (Figure 1). The most recent Surveillance, Epidemiology, and End Results 9 (SEER 9) registry data reflects a therapeutic survival plateau in EWS over the last 20 years (Figure 2), likely owing to the rarity of the disease and a consequent lack of study [3]. For example, from 1993 to 2009, the COG performed 14 phase III trials for acute lymphoblastic leukemia but only 3 for EWS [3–6]. This reflects the limitations of conventional cytotoxic chemotherapy and underscores the need for targeted therapy in EWS. The classic example of a targeted treatment is imatinib therapy in chronic myelogenous leukemia where *bcr-abl*, the Philadelphia chromosome translocation drives tumorigenesis. Directed therapy using imatinib induces dramatic and often durable clinical responses even at doses well below the maximum tolerated dose. Contrastingly, many solid tumors do not possess a clear cut tumor driver. However, Ewing's sarcoma with its specific translocation EWS-FLI1 (11 :  22) opens up a possibility of targeted therapies. So far, it has remained a targetable disease without a targeted drug. 

 Insulin-like growth factor 1 receptor (IGF1R) inhibitors have demonstrated significant activity in EWS [7–9], renewing hope for patients with this disease. However, most EWS patients whose disease responds to IGF1R inhibitors develop resistance to the therapy and disease relapse or recurrence within several months. There are concerns about how to translate these findings of responses of IGF1R responses in EWS into frontline, noncardiotoxic therapy for EWS. Investigations of other novel treatments are clearly warranted. The IGF1R resistance pathways and mechanisms of resistance remain subjects of active research [10]. 

 Unlike other sarcomas, which arise from the mesoderm, the exact cell of origin in EWS is unknown. Because EWS shares some markers with primitive cells of neural lineage, the disease may arise from primitive neuroectodermal cells; however, because EWS most commonly seems to arise from bone tissue, it is also possible that it is of a mesodermal origin [11, 12]. EWS may also arise from a marrow mesenchymal stem cell (MSC) precursor. This is an attractive possibility because MSC may not only be uniquely susceptible to EWS/FLI1 action but also be a source of CD133+ tumor stem cells and high aldehyde dehydrogenase levels [13]. 

 EWS characteristically possesses a unique translocation that results from the fusion of the N-terminal of the EWS gene (*EWS*) on chromosome 22 to the C-terminal of an erythroblastosis virus-transforming sequence-1 (*ETS*) fusion partner. The friend leukemia integration-1 (FLI-1) site accounts for about 85% of fusion transcripts; less commonly, the ETS-related gene (*ERG*), which is located in chromosome 22, is involved [14]. EWS fusion proteins act as aberrant transcriptional regulators and probably cause the critical events that produce EWS transformation [13]. 

 Although different exon-intron combinations are possible, the two most common fusions are either EWS exon 7 to FLI-1 exon 6 (type 1; 51% of EWS patients) or EWS exon 7 to FLI1 exon 5 (type 2; 27% of patients). Two prospective studies have shown that EWS patients with type 1 or type 2 translocations who are given standard chemotherapy have similar outcomes [15, 16]. 

 Since the EWS-FLI1 target is present only in EWS tumor cells and absent in normal cells, directly targeting the action of this abnormal protein is a logical step in the development of a specific EWS therapy. Reduction of EWS-FLI1 expression in cell lines and nude mouse models by nanoparticle-delivered oligodeoxynucleotides, antisense RNA, and siRNA is associated with anti-EWS activity [17, 18]. Although these findings confirm that specific EWS-FLI1 targeting is possible and affects EWS oncogenesis, these laboratory methods are currently too difficult to translate into in vivo approaches in humans. 

 One approach for EWS-FLI1 targeted therapy would be to develop protein-protein inhibitors, a new class of drugs. Recently, surface plasmon resonance screening revealed that YK-4-279, a lead compound with potent anti-EWS activity, blocked RNA helicase A binding to EWS-FLI1, induced apoptosis in EWS cell lines, and reduced growth in EWS xenografts [19]. Since this small molecule is hydrophobic, it should be orally bioavailable and may be suitable for continuous dosing, an important schedule for molecularly targeted agents. 

 This paper will highlight some of the unique opportunities to use new biologic agents to improve outcomes in EWS patients. The successful application of this information in high-risk EWS patients with relapsed or metastatic disease may provide a model for improving the treatment of sarcomas in general.

## 2. Current Status of IGF1R and EWS

**Figure 3:**
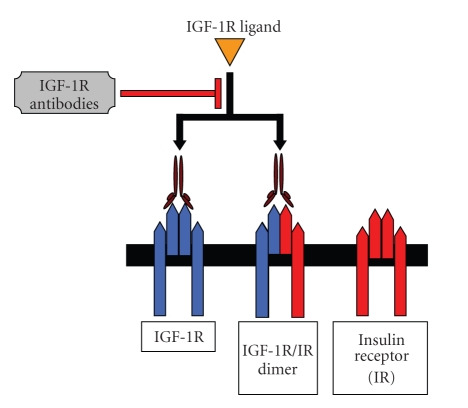
Insulin-like growth factor 1 receptor (IGF1R) system. Ligands (IGF-I, IGF-II, and insulin) bind to the receptors (IGF1R, IGF-2R, and insulin receptor [IR]) with different affinity. The IGF1R and IR possess tyrosine kinase activity. Binding of the IGF-1 ligand to IGF1R leads to a conformational modification of the receptor and activation of the tyrosine kinase subunit. Each receptor triggers complex and different intracellular signaling cascades.

The IGF1R (Figure 3) plays an important role in the growth and development of normal tissue as well as the initiation, maintenance, survival, progression, and metastasis of many sarcomas including EWS [20–22]. The activity of IGF1R in EWS was first demonstrated more than 20 years ago. Additional preclinical studies since then have shown that inhibiting IGF1R suppresses growth in EWS cell lines and EWS xenografts [10]. The introduction of humanized monoclonal antibodies that inhibit IGF1R in phase I and II clinical trials and the dramatic single-agent anti-IGF1R activity observed in refractory EWS patients provided the initial excitement in the sarcoma community [23]. 

 Currently, more than 25 agents acting via IGF1R inhibition are in preclinical and clinical development (Tables 1 and 2). The first of these monoclonal antibodies shown to have activity in EWS was R1507. This finding was initially presented at the 2007 CTOS meeting [23] and prompted the Sarcoma Alliance for Research through Collaboration 011 study, a phase II study that enrolled more than 300 patients with various sarcomas including EWS. Other studies of some of these antibodies have been completed, and studies of R1507, CP-751,871 (figitumumab), and AMG-479 have been published [7–9]. A study of SCH-717454 (robatumumab) was presented at the 2008 Annual CTOS Meeting; this study included a cohort of patients with refractory or resistant EWS as well as two osteosarcoma cohorts [24]. 

 Small-molecule inhibitors of IGF1R are also in preclinical or clinical development (Table 2). In addition to blocking IGF1R, some of these IGF1R inhibitors may also inhibit insulin receptor A, and several have been shown to have promising preclinical EWS activity. These molecules may act more proximal with regard to IGF1R signalling and thus enable oral dosing. On the downside, such agents may have more toxicity than IGF1R monoclonal antibodies.

## 3. Toxicity of Anti-IGF1R Antibodies

IGF1R-targeted monoclonal antibodies have a less toxic safety profile and a higher patient acceptance than currently available cytotoxic chemotherapy regimens for EWS [7–9, 24]. Grade 3 (severe) and grade 4 (life-threatening) events in EWS patients taking IGF1R-targeted monoclonal antibodies are rare (Table 3). Grade 3 hyperglycemia, a concern when using agents that could affect not only IGF1R but also insulin receptor, have been reported but are very uncommon, affecting <5% of patients. Because insulin may drive tumor proliferation, this finding needs to be investigated in patients with frank diabetes mellitus. On the other hand, diabetic patients who receive metformin may benefit from the drug's inhibition of mammalian target of rapamycin (mTOR). Other grade 1 or 2 toxicities such as lymphopenia and thrombocytopenia are also commonly observed when anti-IGF1R antibodies are used and are increased when anti-IGF1R antibodies are used in combination with mTOR inhibitors. 

 Although IGF1R agents have the potential for cardiotoxicity, none of these antibodies have yet demonstrated cardiotoxicity, even in sarcoma patients who received prior anthracycline-based regimens [7–9, 24]. A favorable safety profile of anti-IGF1R antibodies and modest activity has been seen in EWS patients.

## 4. Future Challenges in Developing IGF1R Inhibitors in EWS

Several anti-IGF1R agents in combination with cytotoxic and other targeted agents are currently in clinical development for more common cancers such as lung and colorectal cancer. In theory, however, EWS patients would benefit most from these monoclonal antibodies because EWS has the highest anti-IGF1R single agent activity. 

 Correlative lab and biomarker findings from anti-IGF1R studies may help illuminate the cell signaling and biology of sarcomas in general and EWS in particular. Given that a minority of EWS patients respond to antibody therapy, identifying EWS patients who will benefit most remains challenging. Even if such patients are identified, it will be difficult to convince pharmaceutical companies and regulatory agencies to use clinical trial designs that do not require a large number of patients (e.g., trials in which patients serve as their own controls). Hopefully, serum sample analysis, examination of existing tumor samples, and analysis of tumor biopsies from EWS patients enrolled in clinical studies will help identify which proliferation and resistance pathways should be targeted to achieve the best antitumor response. 

 There are several significant differences in the IGF1R system between mice and humans [37]. For example, mice and humans both express IGF2-P0 transcripts during fetal development; however, IGF2-P0 is not expressed in adult mice but is expressed in humans at all ages [38]. In addition, an inactivated insulin receptor gene is associated with normal growth in mice, but mutations or deletions of the insulin receptor gene in humans with Donohue syndrome have been associated with abnormal growth, resulting in short stature [39]. Such genetic variation may slow biomarker discovery, prediction and validation.

## 5. Rationale for Targeted Drugs Other Than IGF1R in Patients with Refractory EWS

A therapeutic plateau seems to have been reached in EWS despite the use of diverse multidrug chemotherapy combinations [3, 14, 40]. In EWS patients with metastatic or recurrent disease, the outcomes remain dismal, and durable responses are rare. Because IGF1R inhibitors are available only in controlled clinical trials, and because EWS patients develop resistance to IGF1R inhibitors, other options for relapse therapy include enrolling patients in other combinational clinical trials. Trials using mTOR inhibitors or VEGF inhibitors may be one approach. Several options that employ commercially available chemotherapy agents have also been investigated [41] (Tables 4 and 5). Some current and anticipated future novel drug therapies for refractory or recurrent EWS are outlined in Tables 6 and 7. 

 Because no treatment for refractory EWS has been proven to be superior to others, the ideal combination to treat EWS patients with relapsed disease remains unknown. To establish new therapies, it is critical to increase the number of clinical trials offered to EWS patients when their disease first relapses.

## 6. EWS-FLI1 Targeting

**Figure 4:**
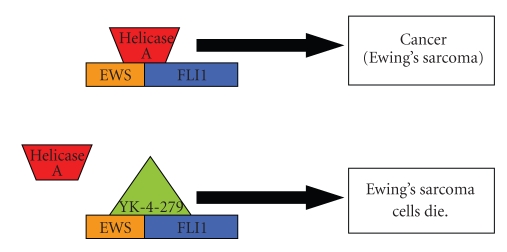
A simplified view of fusion protein: RNA helicase protein disruption, the mechanism of action of a new EWS-FL1 targeted molecule, YK-4-279. Because EWS-FLI1 is a disordered protein that precludes standard structure-based small-molecule inhibitor design, a divergent strategy was designed. EWS-FLI1 interaction with RNA helicase A is critical for oncogenesis. YK-4-279 blocks RHA interaction with EWS-FLI1. This protein–protein inhibition induces apoptosis in EWS cells and reduces the growth of EWS orthotopic xenografts.

Using a small molecule to disrupt key EWS-FLI1 protein-protein interactions may be an effective treatment strategy in EWS patients. In a preclinical model of EWS, a small molecule that blocks the oncogenic protein interaction of EWS-FLI1 with RNA helicase A inhibited tumor growth [19] (Figure 4). This or a similar approach could potentially inhibit EWS oncogenesis in proliferating EWS cells and EWS stem cells in a way that is analogous to imatinib's action against chronic myelogenous leukemia. Although synthesizing and investigating a first-in-class new agent for EWS will be challenging, such an approach may have applications beyond EWS, given that the *EWS* and *ETS* fusion transcripts occur in other sarcomas including desmoplastic small blue round cell tumor (EWS-WTI), myxoid liposarcoma (EWS-CHOP), clear-cell sarcoma (EWS-AFT1), chondrosarcoma (EWS-TEC), and angiomatoid fibrous histiocytoma (EWSR1-ATF1) as well as several other nonsarcomatous cancers including acute myeloid leukemia (TLS-ERG), secretory breast carcinoma (ETV6-NTRK3), and prostate cancer (TMPRSS2-ERG).

## 7. Other Targets in EWS

Given the complex pathways involved in the mechanisms of EWS oncogenesis and drug resistance, new agents will need to be investigated. In the era of genomics and proteomics, potential new pathways will be unravelled to reveal important to host-tumor interactions [56]. Some of the agents targeting these potential pathways are already in different phases of development and are used in other sarcomas and cancers.

1. *mTOR*. mTOR1 pathway signaling may be upregulated when IGF1R is inhibited. Combination treatment is available (Table 6). Another potential benefit of mTOR is that its blockade of IGF1R may prevent the counterproductive rapamycin-induced upregulation of Akt [57]. Although mucositis and/or stomatitis can be a problem with this class of agents, use of glutamine suspension is a simple an effective means to reduce or eliminate this side effect [58, 59].
2. *Phosphoinositide-3 Kinase (PI3K)/Mitogen-Activated Protein Kinase (MAPK)*. The PI3K and MAPK signaling pathways are both constitutively activated in EWS, likely owing to the presence of IGF1R-mediated autocrine loops [11]. Several P13K inhibitors are in different stages of clinical development for other cancers.
3. *Histone Deacetylase*. Histone deacetylase inhibition might inhibit the expression of EWS-FLI1 via the suppression of the EWS promoter activity [60].
4. *Aurora Kinase*. Aurora kinase A is a transcriptional target of EWS. The initial results of a Pediatric Preclinical Testing Program investigation of MLN8237, an Aurora kinase A inhibitor, showed promise for EWS [61].
5. *Hedgehog.* Arsenic trioxide inhibits EWS growth by blocking the Hedgehog/GLI pathway GLI1 both in vitro and in vivo in mouse models [62]. Arsenic trioxide, a useful agent that acts at the level of GLI1 and has already been used to treat acute promyelocytic leukemia, is a potential novel inhibitor of the hedgehog pathway that merits further investigation in EWS [62]. However, given arsenic trioxide's severe cardiotoxicity and neurotoxicity profile, a novel agent such as ZIO-101 (darinaparsin), a small-molecule organic arsenic compound synthesized by conjugating dimethylarsinic acid to glutathione, may be a more reasonable option [63].

## 8. Immunotherapy for EWS

That early lymphocyte recovery (i.e., an absolute lymphocyte count >500 cells/mL on day 15 of the first course of chemotherapy) is a highly significant independent prognostic indicator for high-risk EWS [64] suggests that immune reconstitution constitutes a novel direction in EWS management. Immune reconstitution as a treatment strategy for EWS could be exploited via lymphocyte-sparing chemotherapy agents; for example, Kushner et al. found that lymphopenia was not observed in patients treated with temozolomide plus irinotecan [65]. Alternatively, nutrients such as glutamine could be used to facilitate the proliferation of lymphocytes, which may also benefit young patients in particular by reducing the severity and duration of mucositis [58, 59, 66–68]. Other strategies that could be used to augment lymphocyte proliferation may include cytokine therapy, ex vivo cell culture and infusion therapy [69–71]. 

 In addition to IGF1R antibodies, other specific antibodies and cellular immunotherapy strategies may be used to boost the immune system to overcome drug resistance. Specific immunotherapeutic approaches with vaccine therapy and interleukin-2 with or without cellular therapy have been used to treat patients with recurrent sarcomas (NCT00019279), and autologous T-cell transplantation, vaccine therapy, and indinavir has been used to treat patients with metastatic pediatric sarcomas (NCT00019266). Other trials of high-dose immunotherapy have been reviewed elsewhere [72]. Recent studies have shown that EWS cells are highly sensitive to expanded allogeneic natural killer cells, partially through an NKG2D- and DNAM-1–dependent mechanism, and reveal another potential future direction for immunotherapy in EWS [73, 74].

## 9. Future Research Questions

Many questions concerning the biology of EWS and the information necessary to make decisions about targeted therapies for EWS remain.

**EWS Pathogenesis**

- What is the cell of origin of EWS?
- Is chromosomal translocation the initial event in sarcomagenesis in EWS?
- Does a genetic predisposition lead to this translocation?
- Is EWS-FLI protein action necessary for EWS stem cell survival?
- Is there a genetically relevant preclinical animal model for EWS?

**IGF1R Therapy for EWS**

- What are the active contributions of insulin receptor, IGF1R, insulin-like growth factor-2 receptor, insulin-like growth factor-binding protein-3, and other insulin-like growth factor-binding proteins?
- What are the causes of heterogeneity in clinical response?
- What mechanisms of resistance and biomarker validation could be used to predict response and relapse after IGF1R blockade?
- Can the pathways controlled by IGF1R be validated in clinical trials?
- How can a targeted strategy be combined with current and novel therapy regimens?

## 10. Conclusion

We have come a long way in understanding EWS since the identification of the EWS-FLI-1 translocation. IGF1R-targeted therapies have shown clear benefit in select EWS patients, and it is important to identify the subsets of patients that are most likely to respond to IGF1R-targeted therapy. Now is an exciting time to exploit additional novel therapeutics in sarcomas by optimizing the IGF1R blockade. With a clear sense of the challenges at hand, scientists, clinicians, the National Cancer Institute, the United States Food and Drug Administration, philanthropic foundations, and pharmaceutical companies will have to make a coordinated effort to develop effective new targeted treatments of EWS. The successful translation of EWS targeted therapies into the clinic can then become a model system for a larger number of rare and common translocation-associated cancers. 

## Figures and Tables

**Table 1:** Insulin-like growth factor 1 receptor monoclonal antibodies against Ewing's sarcoma.

Drug	Manufacturer	Current status	Reference
R1507	Roche	PC	[9, 25, 26]
CP-751,871 (figitumumab)	Pfizer	PC	[7]
AMG-479	Amgen	C	[8, 27]
SCH-717454 (robatumumab)	Schering-Plough	PC*	[24]
IMC-A12 (cixutumumab)	Imclone	A+	[28]
MK-0646	Merck	A+	[29]
BIIB022	Biogen Idec	A	[30]
AVE-1642	Sanofi-Aventis	A	[31]

*PC: permanently closed; C: closed; A, accruing; A+: accruing combination trials and/or additional future trials in development.

**Table 2:** Small-molecule inhibitors of insulin-like growth factor 1 receptor (IGF1R).

Drug	Manufacturer	Current status	Reference
OSI-906	OSI Pharmaceuticals	In vivo and in vitro activity in EWS, some activity in chondrosarcoma	[32]
BMS-554417	Bristol-Myers Squibb	In vitro activity against EWS	[33]
XL-228	Exelixis	A multitargeted protein kinase inhibitor targeting IGF1R, FGFR1-3, the Aurora kinases, and the ABL, ALK, and SRC family kinases	[34]
INSM-18	Insmed and UCSF	Orally bioavailable small molecule tyrosine kinase inhibitor that has demonstrated selective inhibition of IGF1R and human epidermal growth factor receptor (Her2/Neu).	[21]
GSK1904529A and GSK1838705A	GlaxoSmithKline	In vitro activity in EWS cell lines	[35, 36]

EWS: Ewing's sarcoma; FGFR1-3: fibroblast growth factor receptor 1–3.

**Table 3:** Uncommon (<10%) Grade 3 and 4 toxicities of antiinsulin-like growth factor 1 receptor (IGF1R) antibodies.

Anti-IGF1R antibody	Grade 3 or 4 toxicity	Reference
R1507	Phase I: lymphopenia, thrombocytopenia, adrenal hemorrhage, hyperglycemia, DVT/PE, CVA;phase II: thrombocytopenia, anemia, pain, hyponatremia, hyperglycemia	[9, 26]
CP-751,871 (figitumumab)	Fatigue, pain, hyperglycemia, increased LFTs, proteinuria; with mTOR RAD001 (everolimus): nausea, fatigue, diarrhea, hypophosphatemia, mucositis	[7, 42]
AMG-479	Phase I: thrombocytopenia, hyperglycemia; phase II: thrombocytopenia, anemia, pain, dyspnea, nausea/vomiting, hyperglycemia	[8, 27]
SCH-717454 (robatumumab)	Constipation, hyperglycemia, back pain	[24]
IMC-A12 (cixutumumab)	With mTOR inhibitor (temsirolimus): hypercholesterolemia, hypertriglyceridemia, hyperglycemia, mucositis (all of these events can be ascribed at least in part to temsirolimus)	[28]
MK-0646	Thrombocytopenia, skin rash, hyperglycemia, fatigue, GI bleeding, elevated LFTs, respiratory problems	[29]

DVT: deep venous thrombosis; PE: pulmonary embolism; CVA: cerebrovascular accident; LFT: liver function test; mTOR: mammalian target of rapamycin; GI: gastrointestinal.

Note**:** Grade 3 or 4 toxicities have been seen in <10% of patients. These antibodies have generally been very well tolerated with few side effects compared to standard EWS chemotherapy.

**Table 4:** Sarcoma chemotherapy combinations with activity in relapsed sarcomas including Ewing's family of tumors.

Drug	Reference
Temozolomide/irinotecan:	[41, 43–45]
Topotecan/cyclophosphamide	[14, 43]
Gemcitabine/docetaxel	[46]
Oral cyclophosphamide/vinorelbine	[47]
Ifosfamide+Mesna	[48–50]

**Table 5:** Biologic agents with potential synergy against Ewing's sarcoma (EWS).

Drug	Comments	Reference
Bisphosphonates	Zoledronate, a potent inhibitor of EWS cell growth in vitro	[51]
Metformin	Metformin inhibits both the mTORC1 pathwayand the IGF1R/IRS-1 pathway and at the same time downregulates the phosphorylation of Akt onserine 473	[52, 53]
Anti-angiogenic agents (bevacizumab)	Preclinical studies: VEGF inhibition suppresses EWS growth. Three of five EWS patients had stable disease for >4 months in a phase I study of bevacizumab	[54, 55]

mTOR: mammalian target of rapamycin; IGF1R: insulin-like growth factor 1 receptor; IRS-1: insulin receptor substrates 1; VEGF: vascular endothelial growth factor.

**Table 6:** Current and future trials of targeted therapy for refractory and/or recurrent Ewing's sarcoma (EWS).

Sponsor site	Drugs	Rationale	Comments	PI contact info/clinicaltrials.gov identifier
Imclone/UTMDACC, Wayne State	IMCA12 + temsirolimus	IGF1R + mTOR	Recently completed accrual for expanded EWS cohort at temsirolimus dose higher than that in children	Aung Naing, MD anaing@mdanderson.org NCT00678769
COG	IMCA12 + temsirolimus	IGF1R + mTOR	Phase I study;pediatric patients with recurrent or refractory solid tumors	Maryam Fouladi, MD maryam.fouladi@cchmc.org ADVL0813; NCT00880282
MSKCC/CTEP	IMCA12 + temsirolimus	IGF1R + mTOR	Phase II study in recurrent or refractory soft tissue or bone sarcomas	Robert Maki, MD PhD makir@mskcc.org NCT01016015

PI: principal investigator; UTMDACC: The University of Texas MD Anderson Cancer Center; IGF1R: insulin-like growth factor 1 receptor; mTOR: mammalian target of rapamycin; COG: Children's Oncology Group; MSKCC: Memorial Sloan Kettering Cancer Center; CTEP: Cancer Therapy Evaluation Program.

**Table 7:** Anticipated future trials of targeted therapy for refractory and/or recurrent Ewing's sarcoma (EWS).

Sponsor site	Drugs	Rationale	Comments	PI contact info
Merck/UTMDACC	MK-0646+ MK8669	IGF1R+/− mTOR	“up front” Rx; Phase II in development	Joseph Ludwig, MD jaludwig@mdanderson.org UTMDACC

Georgetown/UTMDACC	YK-4-279	EWS-FLI1: RNA helicase inhibitor	Currently preclinical; clinical at UTMDACC	Jeffery Toretsky, MD jat42@georgetown.edu Pete Anderson MD, PhD pmanders@mdanderson.org Aung Naing, MD anaing@mdanderson.org
